# Hemicerebral atrophy as an unusual central nervous system manifestation of Neurofibromatosis type 1: A case report

**DOI:** 10.1016/j.radcr.2026.08.017

**Published:** 2026-09-07

**Authors:** Bethlehem Abera, Bethelhem Belachew, Hanan Tofik Ahmed, Yared Dejene Tefera, Tinsae Zelalem Amare

**Affiliations:** a Department of Radiology, St Paul Hospital Millennium Medical College, Addis Ababa, Ethiopia; b Department of Pediatrics and Child Health, Addis Ababa University, Addis Ababa, Ethiopia

**Keywords:** Phakomatosis, Neurofibromatosis type 1, Focal areas of signal intensity, Hemicerebral atrophy, Magnetic resonance angiography, Intracranial vasculopathy

## Abstract

Neurofibromatosis type 1 is a widely known phakomatosis with highly variable manifestations. However, its presentation with hemicerebral atrophy resulting from an intracranial vasculopathy is uncommon, yet it poses a major health threat. We hereby report the case of a 6-year-old boy with the clinical features of Neurofibromatosis type 1 and imaging evidence of right internal carotid artery narrowing, with subsequent ipsilateral hemicerebral atrophy, after presenting with progressive left-sided body weakness and epilepsy. This case represents a rare manifestation within the vast imaging spectrum of Neurofibromatosis type 1, highlighting how unpredictable the condition can be.

## Introduction

Neurofibromatosis type 1 (NF1) is a common autosomal dominant disorder with an approximate incidence of 1 in 3,190 live births [1]. It is one of the most common phakomatoses, characterized by its multisystem involvement and a diverse spectrum of clinical presentations with variable degrees of severity [2]. Its CNS manifestations include optic pathway gliomas, progressive sphenoid wing hypoplasia, focal areas of signal intensity (FASI), and various types of vasculopathies [2,3]. FASI is the most common neuroimaging finding in children affected by NF1, with more than 80% having one or more non-enhancing T2/FLAIR hyperintense lesions involving the basal ganglia, brainstem, cerebellum, and/or thalami [4]. Hemicerebral atrophy, however, to the best of our knowledge, is an exceedingly atypical phenotype in NF1, with only a few reported cases in the literature. The proposed mechanisms for this presentation include hypoplasia or stenosis of the internal carotid artery (ICA) with variable involvement of the circle of Willis, leading to chronic ischemia and resultant atrophy of the cerebral hemisphere [[5], [6], [7]]. The realization of such a rare phenomenon is key, as it widens the neuroimaging spectrum of NF1 and aids in vigilant surveillance and timely intervention. Here, we present a case of NF1 with an early childhood presentation of a vasculopathy-related neurologic manifestation instead of the more commonly encountered CNS markers, such as glioma or sphenoid wing hypoplasia.

### Case presentation

**Fig. 1:**
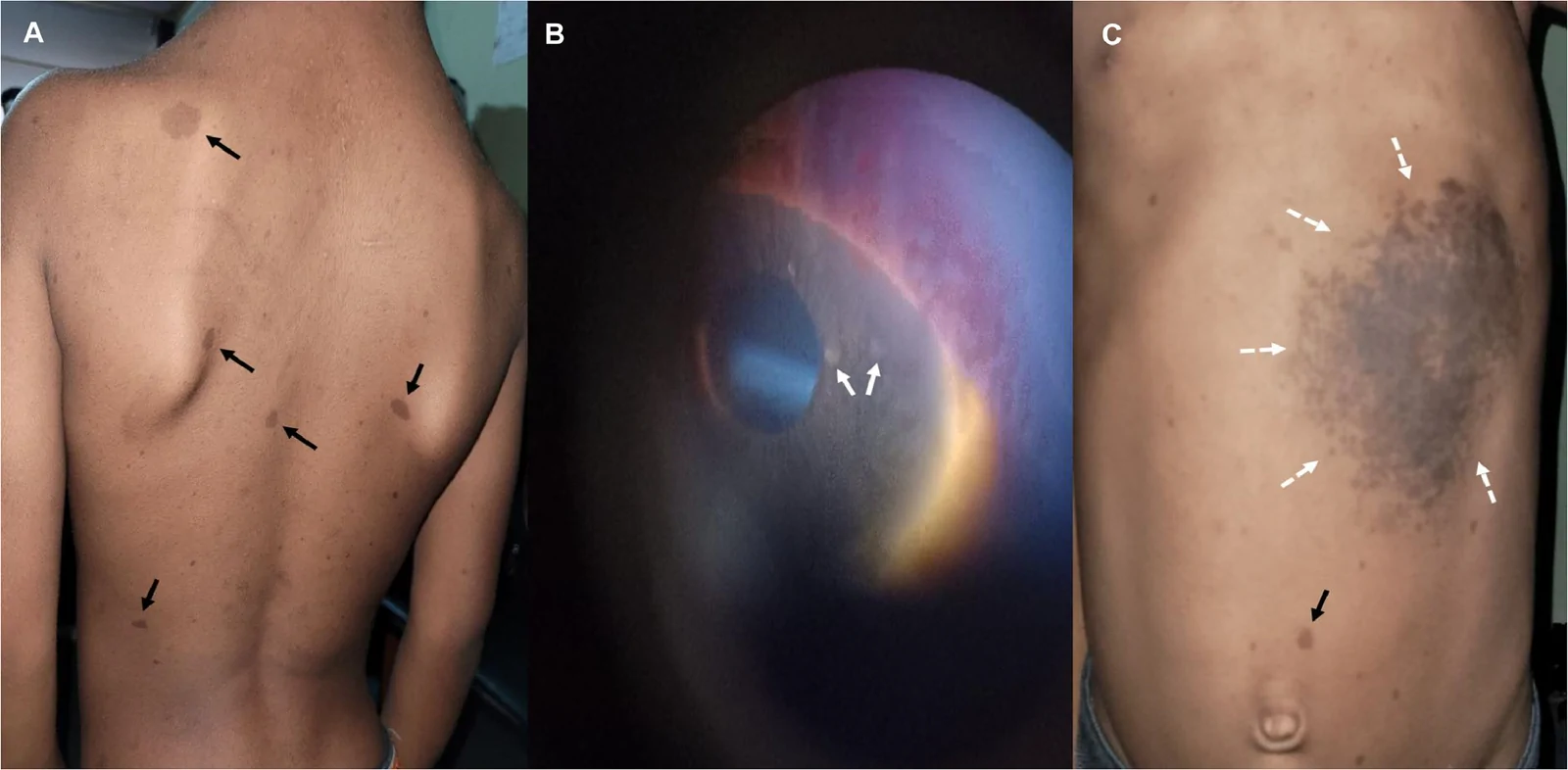
Cutaneous and ocular findings in a 6-year-old boy diagnosed with NF1. (**A)** Multiple café-au-lait macules (*black arrows*). (**B)** Lisch nodules (*white arrows*). (**C)** Dermal plexiform neurofibroma (*dashed arrow*). *NF1,* neurofibromatosis type 1.

A 6-year-old boy presented with a 2-year history of focal seizures, which had recently progressed to status epilepticus, alongside progressive left-sided hemiparesis of 8 months’ duration. Clinical evaluation disclosed features consistent with NF1, including café-au-lait macules, dermal plexiform neurofibromas, and multiple Lisch nodules (Fig. 1). There was no reported family history of the condition, suggesting a sporadic variant, and genetic testing was not carried out due to financial constraints.

**Fig. 2:**
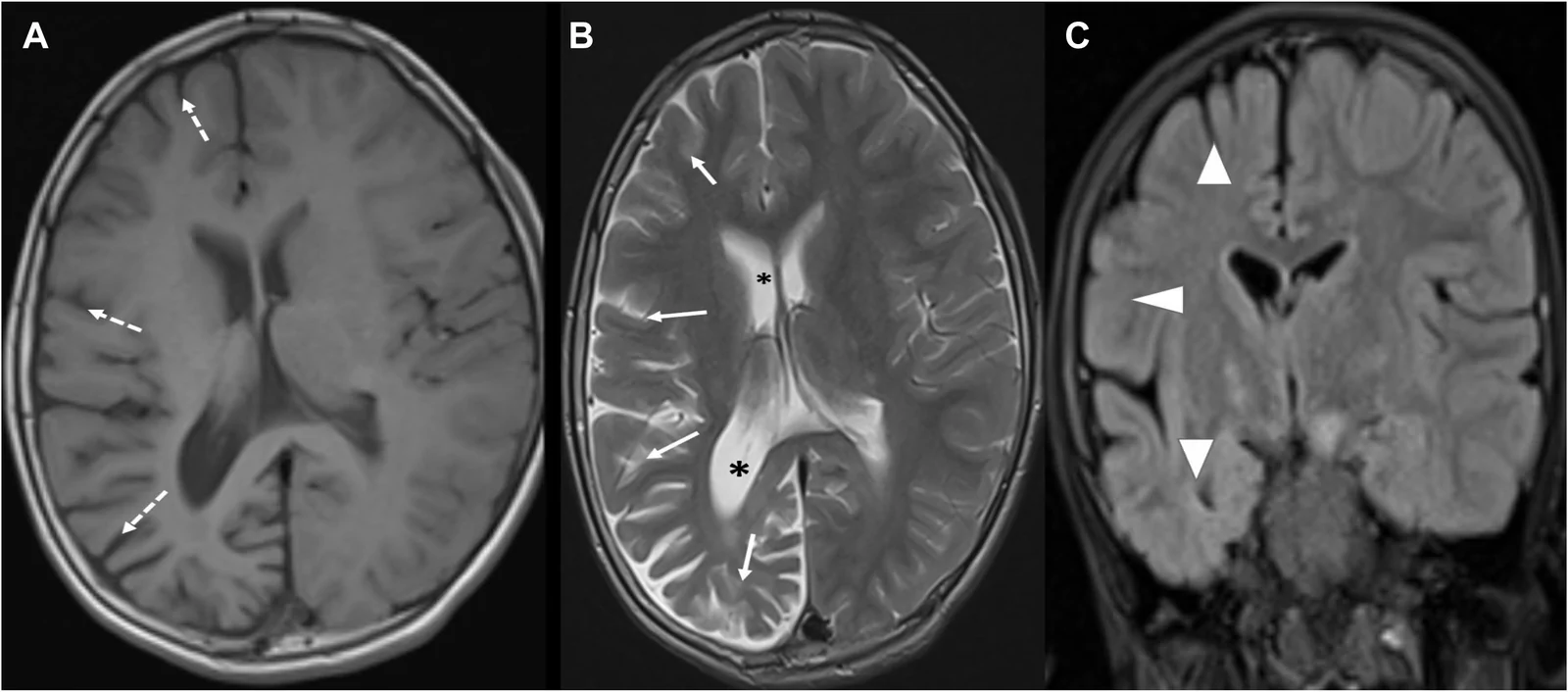
Non-contrast brain MRI shows right hemicerebral atrophy in a 6-year-old boy with NF1. (**A)** Axial T1-weighted image shows right cerebral hemisphere preserved gray-white matter differentiation with volume loss evidenced by asymmetrically widened sulci (*dashed white arrows*). (**B)** Axial T2-weighted image shows right hemispheric atrophy (*solid white arrows*) with associated ex-vacuo dilation of the extra-axial space and ipsilateral lateral ventricle (*asterisk*). (**C)** Coronal FLAIR shows the asymmetry in the cerebral hemispheres (*arrow heads*). *MRI,* magnetic resonance imaging; *NF1,* neurofibromatosis type 1.

Magnetic resonance imaging (MRI) of the brain demonstrated asymmetric right cerebral hemispheric volume loss involving both cortical and subcortical structures, with associated gliotic changes and ex-vacuo dilation of the ipsilateral lateral ventricle as well as prominence of the overlying extra-axial spaces (Fig. 2).

**Fig. 3:**
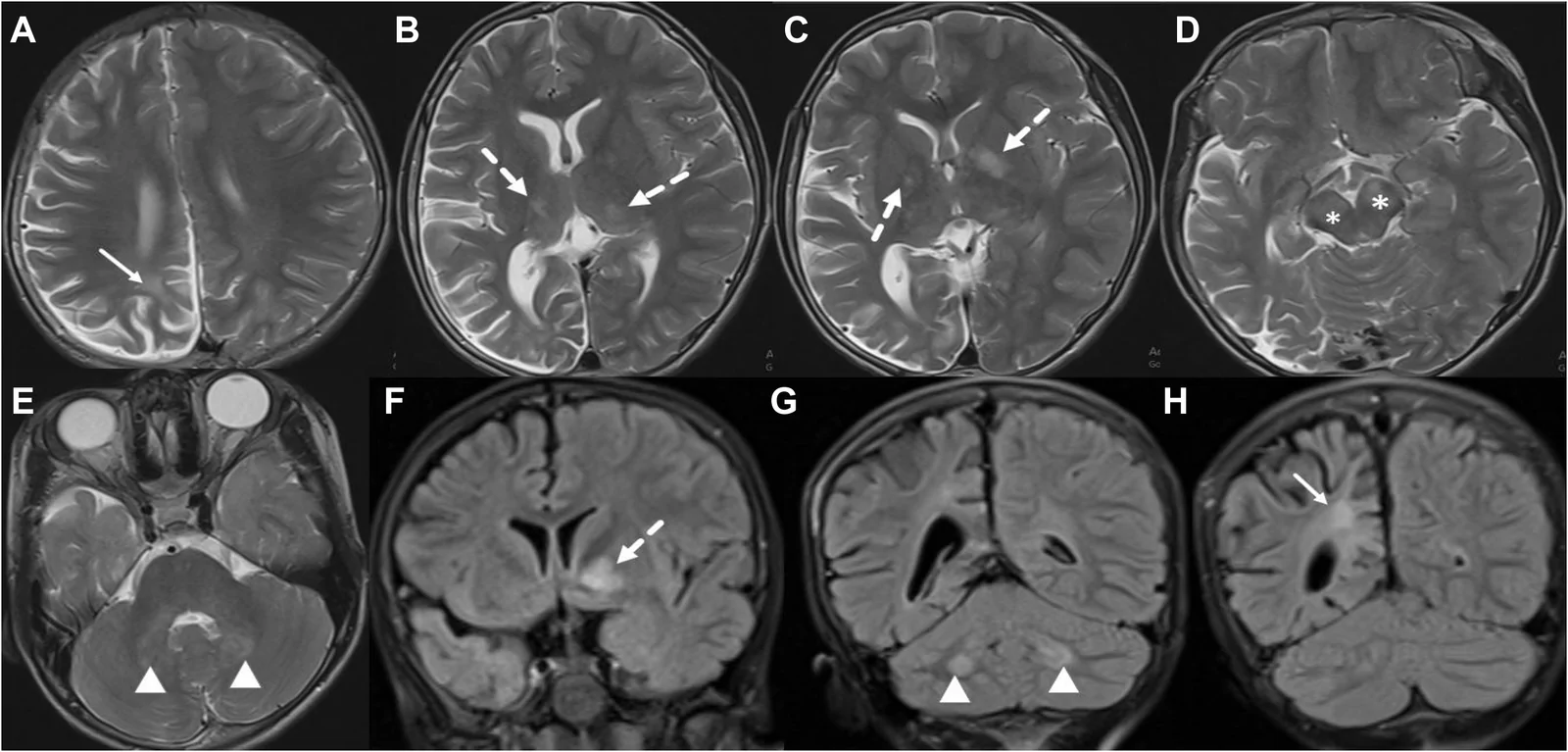
Non-contrast brain MRI of a 6-year-old boy with NF1 (**A–H**) shows axial T2/coronal FLAIR hyperintensities involving the deep white matter (*solid arrows*), deep gray nuclei, and thalami (*dashed arrows*), as well as the midbrain (*asterisk*) and dentate nuclei (*arrowheads*). *MRI,* magnetic resonance imaging; *NF1,* neurofibromatosis type 1.

Multiple FASI were observed as T2/FLAIR hyperintense, non-enhancing lesions (Fig. 3) within the periventricular as well as deep white matter, lentiform nuclei, thalami, brain stem (anterior midbrain, pons, and medulla), cerebellum (dentate nuclei and middle cerebellar peduncle), and splenium of the corpus callosum. The lesions show no restriction on diffusion weighted imaging and apparent diffusion coefficient (DWI/ADC) mapping or blooming on susceptibility weighted imaging (images not shown).

**Fig. 4:**
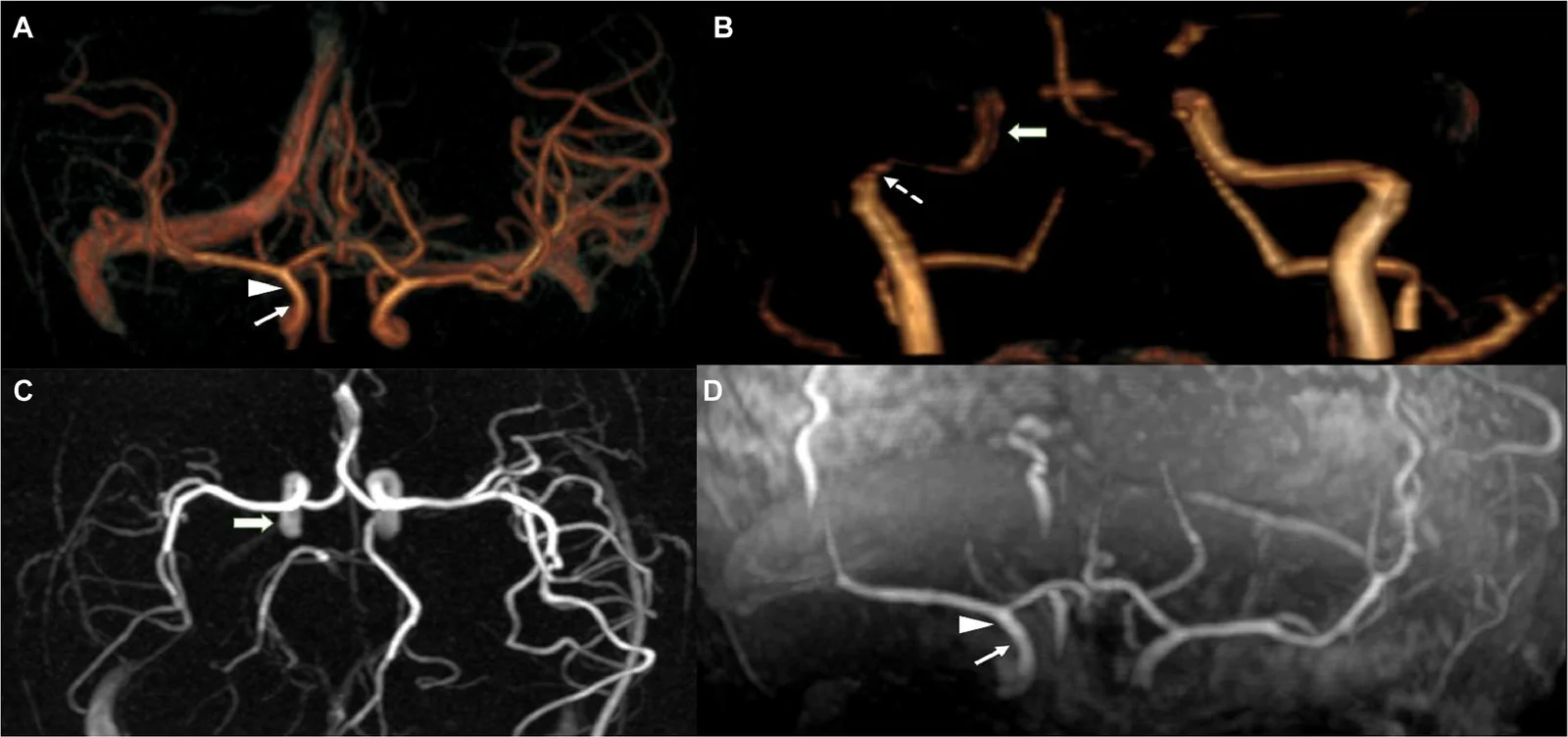
Three-dimensional (3D)-rendered MRI and TOF MRA (**A-D**) in a 6-year-old boy with NF1 show asymmetric long-segment narrowing of the right intracranial ICA, involving the petrous (*dashed arrows*), cavernous (*open arrows*), ophthalmic (*white arrows*), and communicating segments (*white arrowheads*). *ICA,* internal carotid artery; *MRI,* magnetic resonance imaging; *NF1,* neurofibromatosis type 1; *TOF MRA*, time-of-flight magnetic resonance angiography.

Magnetic resonance angiography (MRA) demonstrated a long-segment narrowing of the right ICA compared with the contralateral side (Fig. 4), suggesting an underlying chronic hypoperfusion-related parenchymal change.

The patient is being managed for seizures, maintained on aspirin for stroke prophylaxis, and monitored under surveillance with periodic clinical and imaging follow-up.

## Discussion

The CNS manifestations are among the trademark features of NF1, with optic pathway gliomas and FASI representing the dominant occurrences [2]. The latter, which are abundantly seen in our patient, are believed to result from spongiform myelinopathy with intramyelinic edema and no obvious demyelination [8]. These focal areas of signal abnormality usually appear within a patient’s first year of life, increase in number and size during the first decade—which explains their multiplicity in our case—before spontaneously involuting in late adolescence [4].

NF1-associated vasculopathy is an uncommon but well-documented entity presenting with either aneurysmal dilation or narrowing. Within its vasculopathy spectrum, NF1 can manifest as a Moyamoya syndrome, which is often radiologically indistinguishable from idiopathic Moyamoya disease [9]. However, unlike idiopathic Moyamoya disease—which typically presents bilaterally and has a 2:1 female predilection—NF1-associated Moyamoya syndrome shows an equal gender distribution and a high prevalence of unilateral disease [9]. Hemicerebral atrophy as a predominant presentation, as in our patient, is an even rarer finding in NF1 [3], with only a handful of cases reported in our literature review. The underlying etiology remains uncertain, with potential causes including chronic hypoperfusion from either progressive cerebral vascular stenosis or congenital hypoplasia [[5], [6], [7]]. Given our patient’s later age of presentation, progressive vascular stenosis appears to be the more likely explanation.

The etiologies for hemicerebral atrophy are diverse. In our case, the lack of leptomeningeal enhancement or tram-track calcification makes Sturge–Weber syndrome an unlikely candidate [7]. Rasmussen encephalitis and hemiconvulsion-hemiplegia-epilepsy syndrome can certainly explain the cerebral atrophy. However, these diagnoses are less convincing in our patient due to the presence of classic NF1 clinical features—including café-au-lait macules, dermal plexiform neurofibromas, and multiple Lisch nodules—as well as characteristic FASI [7]. Furthermore, the absence of associated facial atrophy rules out conditions such as Parry–Romberg syndrome [7]. Ultimately, the arterial hypoplasia/stenosis observed in our case, along with the clinical presentation and FASI, establishes NF1 as the standout underlying cause.

As uncommon as arteriopathy is in NF1, it has the potential to culminate in stroke, leading to major debilitation or even death [10]. Early recognition of subtle hemispheric asymmetry, as seen in our patient, is key to investigating the underlying cause and avoiding common misidentification. Its rarity, however, can pose a significant hurdle in diagnosis; this may inadvertently urge clinicians and radiologists to focus solely on more obvious differentials for hemicerebral atrophy, thereby giving the underlying vasculopathy time to progress.

The treatment of NF1-associated cerebral vasculopathy lacks a definite clinical consensus, particularly regarding non-Moyamoya-related entities [3,11]. Medical interventions, such as antiplatelet agents like aspirin, help minimize the risk of platelet-rich clot formation in high-flow stenotic arteries [3]. However, while expert consensus advocates antiplatelet utilization, evidence validating its use for primary prevention remains deficient [11]. In patients manifesting NF1-associated Moyamoya syndrome, surgical revascularization can significantly lower the risk of stroke when performed prior to onset of symptoms. Long-term follow-up shows that surgery nearly completely eliminates recurrent ischemic strokes, though patients face a gradually cumulative risk of *de novo* hemorrhages [11].

Although hypertension is a major risk factor that intensifies the vascular complications of NF1-associated vasculopathy—including stroke—it can be deliberately induced as a rescue management strategy in patients experiencing acute hemodynamic compromise from severe stenosis [11]. Conversely, during chronic phases, target blood pressure levels and management guidelines are not specifically defined for these patient groups; consequently, clinicians tend to rely on standard approaches used for intracranial atheromatous conditions, despite the hereditary etiology of NF1 [11]. Finally, adequate seizure control is of paramount significance, especially in patients with Moyamoya syndrome, as seizures can precipitate the progression from transient ischemic attack (TIA) to a full-blown stroke due to hypoperfusion [12].

## Conclusion

This case strongly underscores the need for careful scrutiny of the brain and its vasculature in NF1 patients presenting with slowly progressive hemicerebral atrophy in the absence of more commonly encountered CNS manifestations, ensuring that timely intervention can be planned. Given the serious consequences of a delayed diagnosis, MRA plays an integral role in surveillance screening for affected patients and should be integrated as a routine diagnostic tool.

## Ethical approval

According to the review board policy of our institution, ethics approval was not necessary.

## Patient consent

Informed written consent was obtained from the patient’s family for publication of this case report and associated images.
